# RETRACTION: Impact of Negative Pressure Wound Drainage Compared With Natural Wound Drainage after Thyroid Tumour Surgery: A Meta‐Analysis

**DOI:** 10.1111/iwj.70503

**Published:** 2025-04-18

## Abstract

**RETRACTION:** ImamM. S. , Abdel‐SattarR. M. , AlamriA. S. , AlqurashiA. M. , AlnefaieA. M. A. , and AbdelrahimM. E. A. , “Impact of Negative Pressure Wound Drainage Compared With Natural Wound Drainage after Thyroid Tumour Surgery: A Meta‐Analysis,” International Wound Journal 20, no. 4 (2023): 1183–1190, 10.1111/iwj.13977.36251756 PMC10031204

The above article, published online on 17 October 2022, in Wiley Online Library (http://onlinelibrary.wiley.com/), has been retracted by agreement between the journal Editor in Chief, Professor Keith Harding; and John Wiley & Sons Ltd. Following an investigation by the publisher, all parties have concluded that this article was accepted solely on the basis of a compromised peer review process. In addition, the investigation found significant textual overlap between this article and another article by different authors [1]. The editors have therefore decided to retract the article. The authors disagree with the retraction.

Reference

[1] LiL. , LiuW. , TaoH. , ChenH. , LiW. , HuangT. , and ZhaoE. , “Efficacy and Safety of Negative Pressure versus Natural Drainage after Thyroid Surgery,” Medicine 97, no. 31 (2018): e11576, 10.1097/MD.0000000000011576.30075525 PMC6081074

**RETRACTION:** M. S. Imam , R. M. Abdel‐Sattar , A. S. Alamri , A. M. Alqurashi , A. M. A. Alnefaie , and M. E. A. Abdelrahim , “Impact of Negative Pressure Wound Drainage Compared With Natural Wound Drainage after Thyroid Tumour Surgery: A Meta‐Analysis,” International Wound Journal 20, no. 4 (2023): 1183–1190, 10.1111/iwj.13977.36251756 PMC10031204

The above article, published online on 17 October 2022, in Wiley Online Library (http://onlinelibrary.wiley.com/), has been retracted by agreement between the journal Editor in Chief, Professor Keith Harding; and John Wiley & Sons Ltd. Following an investigation by the publisher, all parties have concluded that this article was accepted solely on the basis of a compromised peer review process. In addition, the investigation found significant textual overlap between this article and another article by different authors [1]. The editors have therefore decided to retract the article. The authors disagree with the retraction.

**Reference**

[1] L. Li , W. Liu , H. Tao , H. Chen , W. Li , T. Huang , and E. Zhao , “Efficacy and Safety of Negative Pressure versus Natural Drainage after Thyroid Surgery,” Medicine 97, no. 31 (2018): e11576, 10.1097/MD.0000000000011576.30075525 PMC6081074

